# Solar Photochemistry in Flow

**DOI:** 10.1007/s41061-018-0223-2

**Published:** 2018-11-19

**Authors:** Dario Cambié, Timothy Noël

**Affiliations:** Micro Flow Chemistry and Process Technology, Department of Chemical Engineering and Chemistry, Eindhoven University of Technology, Den Dolech 2, 5600 MB Eindhoven, The Netherlands

**Keywords:** Solar photochemistry, Flow chemistry, Green chemistry, Solar energy

## Abstract

In recent years, photochemistry has been a highly active research field. This renaissance is linked to the upsurge of photoredox catalysis, a versatile platform for synthetic methodologies using visible light photons as a traceless reagent. In contrast with UV, visible light constitutes almost half of the ground solar irradiance, making the use of solar light in chemistry a sustainable and viable possibility. However, the direct use of sunlight to power chemical reactions is still little explored. This can be explained by both the hurdles associated with solar radiation (e.g., its variability, irreproducibility, high IR content, etc.) and the need for a specialized photoreactor. Most of these issues can be tackled with technological solutions, and especially with the recourse to flow chemistry. Flow chemistry goes hand in hand with photochemistry thanks to the uniform irradiation it provides to the reaction. Furthermore, a continuous-flow reactor can be easily integrated with different solar collectors (including compound parabolic concentrators and luminescent solar concentrators) and constitutes the most efficient approach to solar photochemistry. After a description of the characteristics of the solar radiation relevant to chemistry, this chapter critically describes the different type of solar photoreactors and their applications in synthetic organic chemistry. Finally, an outlook on the future of solar photochemistry in flow is included.

## Introduction

The sun constitutes the most sustainable light source available for photochemistry. However, the use of solar light in photochemistry comes with significant hurdles associated with its polychromatic nature and fluctuating ground intensity. These limitations, coupled with problematic access to natural sunlight in a laboratory setting and the safety issues connected with the use of chemicals outdoors, constitute significant entry barriers to the chemists willing to step into the solar photochemistry arena.

Notably, none of those restraints are of a fundamental nature, therefore, technical and technological solutions can be devised to avoid or circumvent these issues. For example, the commercial availability of artificial lamps that accurately mimic the characteristics of solar light (the so-called “solar simulators” originally developed for photovoltaics testing [1]) can significantly ease lab investigations on photochemical reactions at high photon fluxes. Similarly, to efficiently use the solar photons and to maintain control over the reaction temperature, several solar photoreactor designs have been developed. In this regard, flow chemistry imposes itself as the ideal solution to efficiently deliver the solar photons to the reaction medium. Flow setups allow for an easier integration of solar collectors, devices often adopted to compensate for the relatively low intensity of the solar irradiance. Furthermore, the adoption of flow also comes with additional benefits in terms of high heat transfer, thanks to the higher surface-to-volume ratio, and simple interface with analytical instruments for both reaction control and automation. It is a natural consequence that the majority of photoreactors specifically designed for solar application are continuous-flow reactors.

In this chapter, the applications of continuous-flow chemistry to organic solar photochemistry will be described. The first section offers a brief historical perspective and a description of the characteristic of the solar irradiance relevant to the chemist. After that, the different solar photoreactor designs will be presented with their characteristics and applications. Finally, given the evident sustainability premises of the field [2], an outlook on the future role of solar photochemistry in the context of a general trend towards greener chemistry solutions will be provided.

## Historical Perspective

At the beginning of photochemistry, the sun was the only light source available [3–5]. Due to its abundance and ease of access, solar light endured as a prime light source for photochemical reactions from the earlier pioneers of the nineteenth century until the beginning of the twentieth century. With the introduction of increasingly cheaper and more powerful artificial light sources, however, the preference of chemists rapidly changed. By 1968, the list of light sources available to photochemists included in the second edition of Schönberg’s “preparative organic photochemistry” only briefly cited sunlight alongside with several remarks on its shortcomings [6]. During the second half of the twentieth century, the use of sunlight in organic photochemistry has mostly remained neglected, with the sole exception of wastewater treatment applications, which are out of the scope of this chapter [7].

More recently, the interest in solar photochemistry has resurged thanks to the increased interest towards greener chemical processes [8]. After the seminal work conducted in the 1990s at the Plataforma Solar de Almería (PSA) and at the German Aerospace Center (Deutsches Zentrum für Luft- und Raumfahrt, DLR) near Cologne [9], in the 2000s several examples of photochemical reactions powered by natural sunlight started to appear in the literature. In most of the cases, though, the use of sunlight did not constitute the main object of research but served merely as a proof over the mildness condition required for reaction activation (i.e., visible light as opposed to UV photochemistry [10]). Among the photoreactors specifically designed for solar applications (i.e., SOLFIN, SOLARIS, PROPHIS, MPI line-focusing, sunflow, and LSC-PM), each of them features a continuous-flow design.

The wide application of flow in solar photochemistry is not fortuitous but constitutes the deliberate choice of maximizing the photon flux received by the reaction mixture. In fact, given the relatively low intensity of the solar irradiance, the efficient use of solar photons is of paramount importance. For this reason, a description of the main parameters affecting the solar radiation at ground is provided in the next section.

## Solar Radiation

### Solar Constant

The extraterrestrial solar spectral irradiance has been intensely studied since the 1960s, mostly because of its importance in satellite-mounted photovoltaics [11]. The standard intensity of the solar extraterrestrial radiation on a unit area exposed normally to the sun rays at one astronomical unit is called the “solar constant”. The value of the solar constant has been a subject of debate in radiometry over the twentieth century, mainly due to the low precision of ground-based instruments. The value of the solar constant as measured in space is about 1366 Wm^−2^ [12]. Despite its name, this value is not constant [13] but slightly fluctuates, due to the variation in solar activity, on every timescale at which it has been measured (from minutes to decades). Moreover, since the Earth’s orbit around the Sun is elliptical, yearly variations in the Sun–Earth distance (about 3%) are also affecting the total solar irradiance.

### Spectral Distribution of the Extraterrestrial Solar Irradiance

**Fig. 1:**
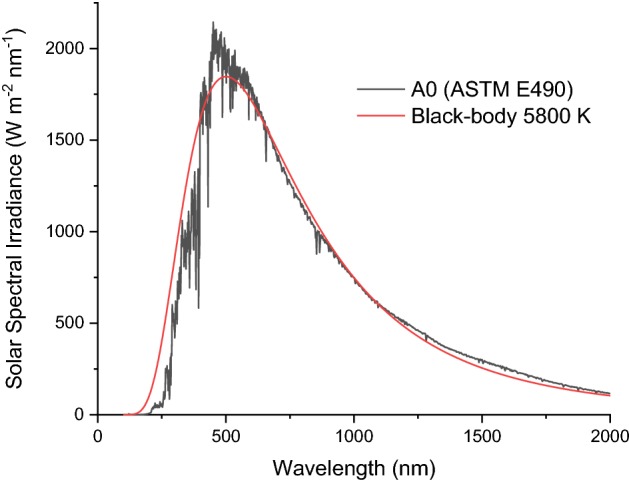
Comparison between the radiometric measurements of the solar spectral irradiance and the calculated blackbody radiation at 5800 K. It can be observed how the solar spectrum does not strictly follow the blackbody law

While nowadays the absolute value of the solar constant is known with high accuracy and precision, larger uncertainties are associated with its spectral distribution. The simplest description of the solar spectral irradiance is obtained from Planck’s law, considering the solar spectrum as a black-body at about 5800 K. While this approach is oversimplified, it provides a reasonable estimation for the fraction of solar spectrum in either UV, visible or IR. Currently, the latest radiometric measurements of the extraterrestrial solar irradiance (also referred to as “zero air mass” solar spectra irradiance) are collected in the ASTM standard E490-00a [12]. From the comparison between the calculated blackbody radiation and the radiometric measurements plotted in Fig. 1, it is evident how the actual spectral irradiance from the sun does not strictly follow the black-body law.

### Spectral Distribution of the Ground Solar Irradiance

Unless the reaction is taking place in outer space (where, incidentally, flow chemistry is a necessity due to the lack of gravity [14]) the impact of the earth atmosphere on the solar radiation has to be taken into account. The simplest description of atmosphere impact is attained in clear sky conditions. Several models of clear sky solar irradiance are available [15]; among them, the Simple Model of the Atmospheric Radiative Transfer of Sunshine (SMARTS) [16], freely available from the National Renewable Energy Laboratory (NREL) is particularly useful, thanks to its versatility. With SMARTS, the solar irradiance intensities and spectral distributions can be calculated for every location and time.

**Fig. 2:**
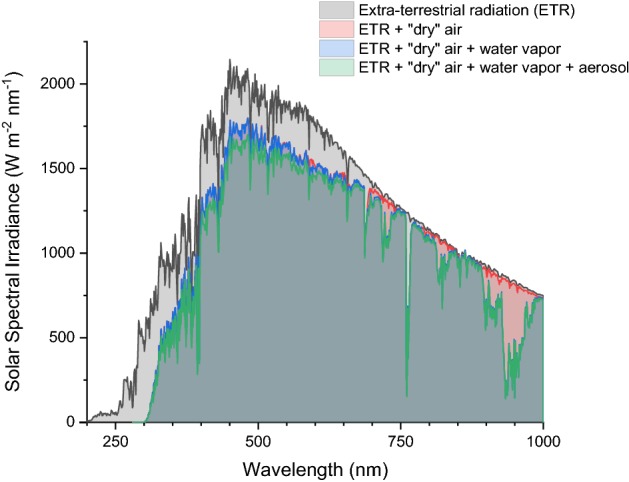
Effect of the earth atmosphere on solar radiation at ground (spectra modeled with “Simple Model for Atmospheric Transmission of Sunshine” SMARTS v. 2.9.5. Absolute air mass 1.5, precipitable water 1.42 cm, ozone 0.34 cm, turbidity at 500 nm 0.084, CO_2_ 370 ppmv)

In cloudless and clear-sky conditions, mainly three components affect the earth atmosphere transmission (in decreasing order of importance): dry air molecules (e.g., nitrogen, oxygen, argon, CO_2_), water vapor and aerosol. Although the distribution of the gasses constituting the atmosphere is not uniform throughout the earth (e.g., the ozone depletion is mainly centered over Antarctica), the variations in atmospheric composition with location, elevation and season are limited. More significant seasonal and location-dependent fluctuation are observed in the precipitable water, i.e., the water contained in a column of unit cross-section extending from the Earth’s surface to the “top” of the atmosphere. Finally, the last atmospheric parameter affecting solar irradiance in cloudless conditions is the aerosol, i.e., the presence of small suspended particles that manifests itself with a reduced visibility or increased turbidity. In Fig. 2, the effect of a standard atmosphere is shown with respect to the extra-terrestrial radiation (ETR). The absorption by the ozone layer is responsible for a significant shielding in the UV-portion, shifting the begin of the window for solar photochemistry from 250 to 300 nm [17].

**Fig. 3:**
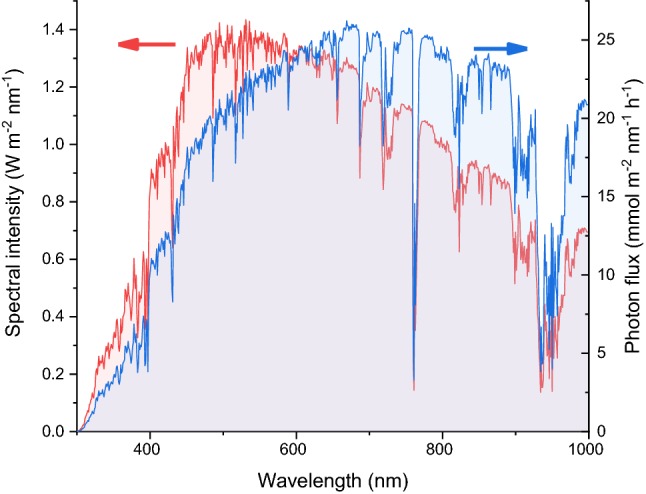
Comparison between the solar spectrum (AM 1.5G) expressed in $W\,{\text{m}}^{- 2}\,{\text{nm}}^{- 1}$ and ${\text{mmol}}\,{\text{m}}^{- 2}\,{\text{nm}}^{- 1}\,{\text{h}}^{- 1}$

The lack of UVC photons in the solar radiation at ground has a significant impact on the photochemical transformation that can be powered by solar light. In recent years, mostly thanks to photoredox catalysis, visible light has been recognized as a viable activation method for several reactions. On the one hand, the use of visible light simplifies the photoreactor design: for example, UV-transparent glass (e.g., quartz of Vycor) can be replaced by less expensive glass or polymeric materials. On the other hand, the wide availability of inexpensive, efficient and narrowband lamps emitting in the visible, such as LEDs, reduces the attractiveness of sunlight as a free photon source. It should be noted, though, that the intensity of the solar spectrum in the visible is significantly higher than in the UV range (see Fig. 2). This comparison is even more compelling when the solar spectrum is described in term of its quantic photon flux as opposed to the energy associated with its radiation. Indeed, for most solar irradiance data, the y-axis represents the intensity of solar energy, usually expressed in watts per square meter. Since photochemical reactions are quantum processes, the photon flux is a more meaningful metric. The photon energy is proportional to its frequency (and therefore inversely proportional to the wavelength) according to the Planck–Einstein relation $E = h\,\nu,$ where $h$ is the Planck’s constant and $\nu$ the photon frequency. Therefore, the energy content of solar radiation can be converted, nanometer per nanometer, in the corresponding photon flux, as shown in Fig. 3.

Notably, when the photon fraction is considered as opposed to the energy content of the solar radiation, the already small fraction of UV photons shrinks even further: only 0.3% of the solar photons at ground have wavelength lower than 350 nm even though, due to their higher energy content, they account for about 1% of the solar irradiance total energy. Despite not being part of the International System of Units, the Einstein (*E*) is a metric often used to express photon molar quantities. In the wavelength range up to 700 nm, the reference solar irradiance at 1.5 air mass (AM 1.5G) contains $6.6 E\,{\text{m}}^{{- 2}}\,{\text{h}}^{{- 1}}$ (see Table 1). For a molecule with a molecular mass is 200, this physical limit for solar photochemistry productivity would translate in 1.3 kg of product synthesized per square meter per hour.

**Table 1:** Comparison between the energy and photon fraction in the UV, VIS and IR portions of the solar spectrum (AM 1.5G)

	Energy content		Photon fraction	
	W·m^−2^	%	Mol·h^−1^·m^−2^	%
280–350 nm (UV)	8	0.9	0.1	0.3
350–700 nm (VIS)	398	44.2	6.5	27.3
700–4000 nm (IR)	495	54.9	17.3	72.4
Total	901	100	23.9	100

For visible-light reactions, high-energy UV photons can be detrimental and might affect the reaction selectivity. Two different strategies can be implemented to shield the reaction for the UV portion of solar light. Either the UV photons are prevented to reach the reaction mixture (by selective reflection or absorption) or they are down-converted to longer wavelengths. The latter strategy is evidently advantageous as it can translate into an increased photon-efficiency and, so far, is unique to the LSC-PM reactor design [18].

### Diffuse Solar Radiation

**Fig. 4:**
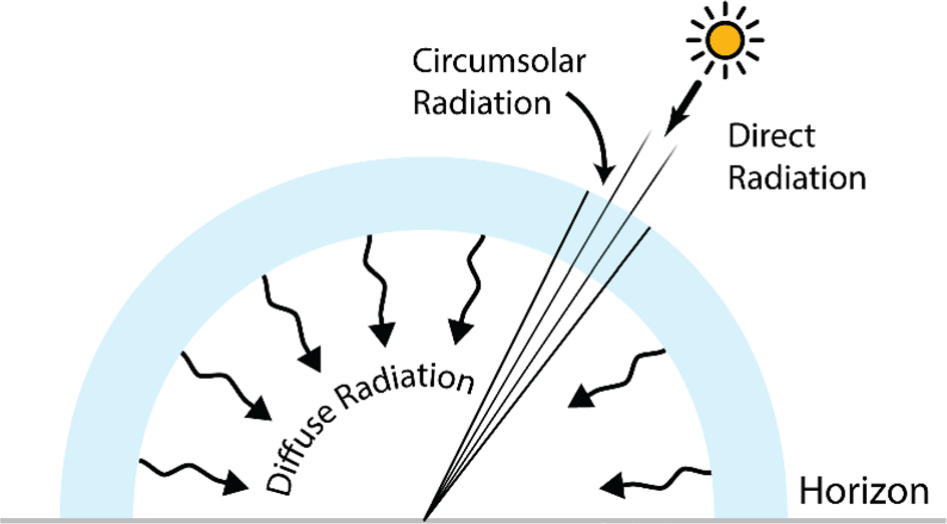
Components of the global solar radiation: direct, circumsolar and diffuse

The solar radiation can be divided into two components: direct (or beam) and diffuse (see Fig. 4). As described earlier, the beam radiation is reduced in intensity by the atmosphere constituents absorption. On top of this, the interaction between the solar photons and other particles can cause scattering phenomena, that are responsible for the characteristic blue color of the clear sky. Based on the size of the interacting particles, both Rayleigh (for air molecules) and Mie (water vapor or dust) scattering can occur. Furthermore, the interaction between the direct solar radiation and clouds can add up to the diffuse component of solar radiation. Finally, multiple scattering and multiple reflections further increase the importance of the diffuse component of solar radiation. As a first approximation, the diffuse component of solar radiation can be considered isotropic (see Fig. 4), even though this is true only when the sky is completely overcast by clouds.

The sum of beam and diffuse components constitutes the global radiation (also known as total radiation), which can be measured with a pyranometer as the sum of all radiation incident on the ground on a 2π solid angle. Similarly, the value of the direct radiation can be obtained with a pyrheliometer, an instrument with a small aperture following the solar disk in the sky. Notably, in this value also a small contribution of forward scattered light (the so-called circumsolar radiation) is included. The knowledge of the fractional contribution of diffuse and beam radiations is important in the design of a solar photoreactor. At higher latitudes, for example, the yearly contribution of diffuse radiation can be larger than its direct counterpart, meaning that parabolic collectors will not be as efficient as expected considering the global radiation values alone. A more in-depth description of solar radiation can be found in the book “An introduction to solar radiation” by Muhammad Iqbal [19].

### Solar Productivity Metrics

Both in the optimization and in the comparison of different solar-powered synthetic processes, it is important to have metrics to characterize the different parameters. In fact, not only the reactor design and the process contribute to the reaction performance but also external variables related to the solar irradiance. When possible, the variability associated with the solar irradiance can be eliminated by comparing different conditions side-by-side, so that the solar irradiance conditions can be neglected. While in this simplified case any metric related to the reaction progress would be suitable, the most appropriate parameter is the molar productivity per unit surface and time (e.g., ${\text{mol/m}}^{ 2}\,{\text{hr}}$) as it allows the comparison between reactors with a different irradiated area.

Often, comparisons between different solar photochemical syntheses performed in different locations are needed. The most convenient parameter, in this case, is the “total photon yield” ($\eta_{g}$) introduced by Scharf and co-workers [17], and defined as follows:

$$\eta_{g} = \frac{{\mathop \smallint \nolimits_{0}^{\lambda } n_{h\nu } \left( \lambda \right)\,\eta_{PR} \left( \lambda \right)\,\eta_{Abs} \left( \lambda \right)\,\phi_{R} \left( \lambda \right) d\lambda }}{{\mathop \smallint \nolimits_{0}^{\lambda } n_{h\nu } \left( \lambda \right) d\lambda }} = \frac{{n_{B} }}{{\mathop \smallint \nolimits_{0}^{\lambda } n_{h\nu } \left( \lambda \right) d\lambda }}$$

where $n_{h\nu }$ is the fraction of solar photons with $\lambda$ wavelength, $\eta_{PR}$ the photoreactor efficiency, $\eta_{Abs}$ the absorption yield, $\phi_{R}$ the reaction quantum yield and $n_{B}$ the moles of product obtained. Usually total photon yield values are reported accounting for wavelength up to 700 nm, defined by Scharf and co-workers as “solar chemical threshold wavelength” [17]. To compare the total photon yield values to other solar technology that report their efficiency over the global radiation, the total photon yield can be divided by two since about half of the solar spectrum is found before the 700 nm threshold.

Notably, the total photon yield combines parameters relevant to the environment ($n_{h\nu }$), the reactor ($\eta_{PR}$) and the reaction ($\phi_{R}$ and $\eta_{Abs}$) to account for the apparent quantum yield of the whole process. As such, this parameter can be used to compare the efficiencies of different reactions under solar irradiation. It also provides a concise yet intuitive representation of all the factors affecting the efficiency of a solar photochemical process. For example, a [2 + 2] photocycloaddition between ethylene and 5-ethoxyfuranone presented a solar photon yield of about 0.1% while a singlet oxygen reaction sensitized by methylene blue offered total photon yields in the 15–20% range [17], thus highlighting the superior suitability of visible-light transformation over UV reaction for solar applications.

Despite its advantages, the total photon yield has not seen wide adoption. This is probably due to the difficulty to measure or estimate all the parameters required and their wavelength dependency. Furthermore, since the moles of product are used as production metric, for non-zero order reaction kinetics, the reaction extent (i.e., the reaction conversion) also affects the total photon yield, meaning that the same process will show lower total photon yield at higher conversion levels, depending on the reaction kinetic profile.

## Reactor Designs

In this section, the flow photoreactors specifically designed to use solar light will be described, including a brief description of their relevant synthetic applications. Almost all of them make use of solar concentrating devices to increase the photon flux toward the irradiated capillary or tube. The only exception in this regard is constituted by the Sunflow reactor [20], that instead employed a long capillary (25 m of FEP) to increase the solar-collecting area.

### Solfin

**Fig. 5:**
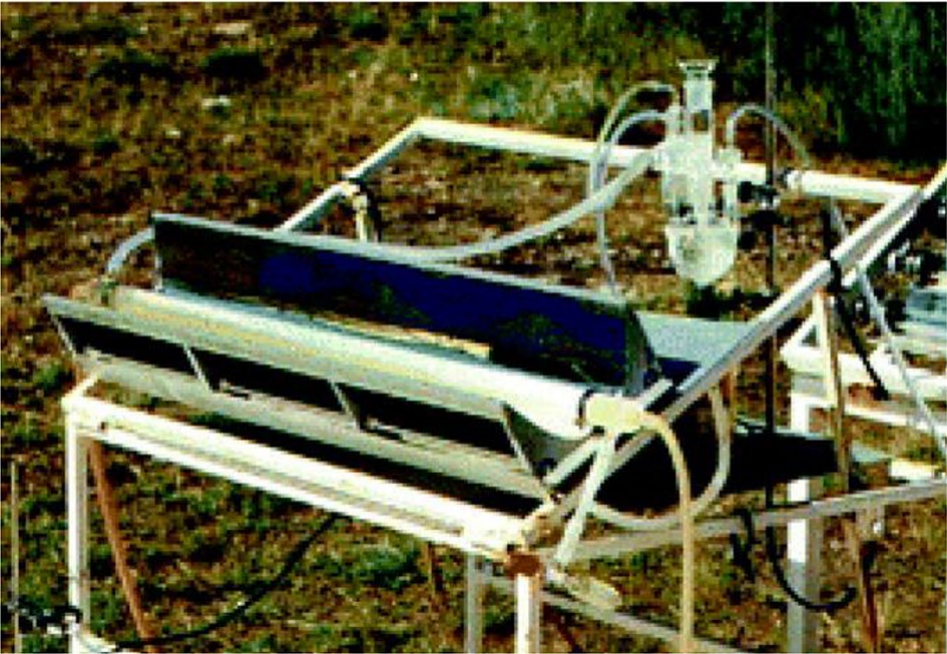
The single-loop version of the SOLFIN reactor. The liquid condenser placed in the focus of the parabola is connected to both the water cooling and the reaction mixture reservoir. Reprinted with permission from [23]. Copyright 2009 The Royal Society of Chemistry

The SOLFIN (SOLar synthesis of FINe chemicals) facility hosts two compound parabolic concentrators (CPC) reactors built between 1996 and 1997 at the Plataforma Solar De Almería, in Spain [21]. The first reactor is constituted by an array of eight 48-mm tubes placed in the focus of a 152 mm wide and 1-m long CPC-collector with aluminum reflectors. To increase the photon flux directed toward the reactor, the unit is tilted south 35° to compensate for the site latitude. Given that the acceptance angle of the CPC is about 60° on either side of the normal, it was claimed that not only direct light, but also a good fraction of diffuse sunlight is directed towards the tube. Another reactor based on a similar design was also built for reactions on a smaller scale, employing a single 32 mm OD Liebig-type glass condenser mounted in the focus of a 1-m long and 20 cm wide reflecting parabola. For both the reactor designs the optical concentration was about 4 [22]. The condenser is operated in the opposite way than usual: the outer layer hosts the recirculating reaction mixture, while on the inside the cooling water maintains the reaction temperature constants (see Fig. 5).

The SOLFIN reactor has been used both for photochemical reaction, including 2 + 2 Paternó-Büchi cycloadditions, photoisomerization and photocyclization, and photocatalytic reaction with TiO_2_ and benzophenone as photocatalysts.

**Scheme 1:**
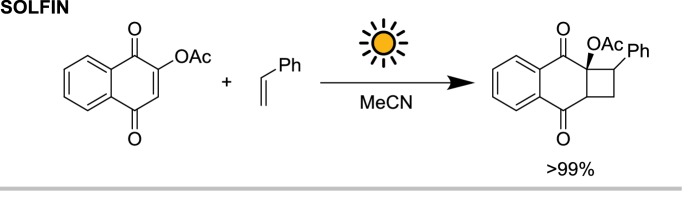
The 2 + 2 photocycloaddition performed in the SOLFIN reactor powered by solar irradiation. Higher selectivity was obtained using solar light as opposed to a 125 W medium pressure mercury lamp

Gilbert and co-workers [24] in 1998 used the single-loop SOLFIN reactor for the 2 + 2 Paternó-Buchi cycloaddition of arylethene with 2-substituted naphthoquinones (Scheme 1). Under solar irradiation, the head-to-head adduct was obtained in a quantitative yield on a 10 g scale (18.5 mmol) after 6 h of irradiation, with a productivity of $15.4 \;{\text{mmol}}\,{\text{m}}^{{- 2}}\,{\text{h}}^{{- 1}}$. Interestingly, no byproducts were observed with reaction temperatures up to 60 °C, meaning that the water cooling capabilities of the SOLFIN were not needed in this case. Due to the low extinction coefficient of the naphthoquinone in the visible range, increasing the substrate concentration up to 6% w/v resulted in an extension of the absorption cut-off (defined as absorbance of 1.5) up to 430 nm, increasing the reaction rate.

**Scheme 2:**
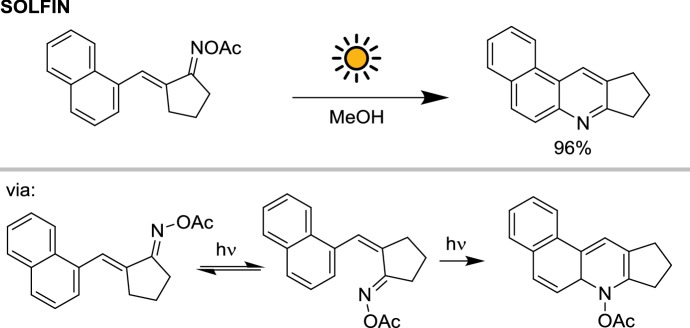
Photoisomerization and photocyclization of 2-napthylidenecyclopentanone oxime *O*-acetate in natural sunlight with the SOLFIN reactor

Another photochemical reaction investigated with the SOLFIN reactor was the cyclization of α,β-unsaturated *O*-acetyloximes [25]. Due to the UV-A absorption of the substrate, the smaller SOLFIN reactor with Pyrex glass was used. After initial *E*,*Z*-photoisomerization at both C–C and C–N double bonds, the E,Z-isomer undergoes a photocyclization to the corresponding dihydroquinoline, followed by rapid elimination of acetate yielding the aromatic quinoline. This means that the process needs at least two photons (*E*,*Z*-isomerization and cyclization) per molecule. A series of *O*-acetyloximes were reacted under solar irradiation. For the naphthyl derivative, 5 g of starting material (18 mmol) were fully converted in 6 h, resulting in the corresponding quinoline in 96% isolated yield (Scheme 2). The reaction productivity, despite the two-photon process, was still about $15 \;{\text{mmol}}\,{\text{m}}^{{- 2}}\,{\text{h}}^{{- 1}}$, comparable with that observed for the 2 + 2 cycloaddition in the previous example.

**Scheme 3:**
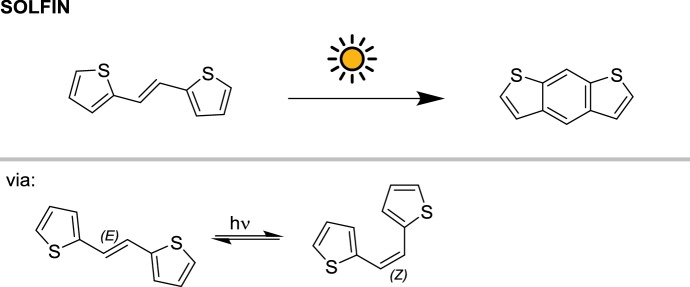
The photochemical cyclization of 1,2 dithiophenylethylene performed in the SOLFIN reactor

Mechanistically similar to the previous example is the photocyclization of 1,2-diheteroarylethylenes to synthesize thiohelicenes (Scheme 3) reported by Caronna and co-workers [26]. In this case, a faster kinetic profile was observed by irradiation with concentrated sunlight: reaction completion was achieved in 2 h as opposed to the 10 h needed in the lab with a Rayonet reactor equipped with 16 lamps (8 W each).

**Scheme 4:**
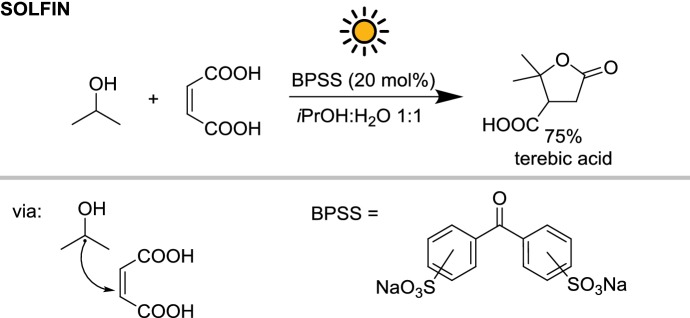
Benzophenone photocatalyzed solar alkylation of electron-poor olefins performed in the SOLFIN photoreactor. A water-soluble photocatalyst is used to simplify the reaction work-up

More recently, the same reactor has been employed by Fagnoni and co-workers for the radical alkylation of α,β-unsaturated acids or aldehydes [23]. The alkyl radicals derived from isopropanol and 1,3-dioxolane were generated under solar irradiation in the presence of disodium benzophenodisulfonate (BPSS), a water-soluble benzophenone derivative, and subsequently trapped by electron-poor olefins (Scheme 4). Unfortunately, the absorption window of this photocatalyst is limited to UV-A (about 360 nm), resulting in an inefficient use of the solar spectrum. For example, 14 h of irradiation over 3 days were needed to obtain 14 g of terebic acid (89 mmol) in 75% isolated yield. This resulted in a productivity of $32 \;{\text{mmol}}\,{\text{m}}^{{- 2}}\,{\text{h}}^{{- 1}}$. However, reaction times were comparable with those obtained with a 125 W mercury lamp. Finally, due to the optically concentrating nature of the SOLFIN reactor, it was observed that in cloudy weather conditions the reaction progress was significantly reduced.

**Scheme 5:**
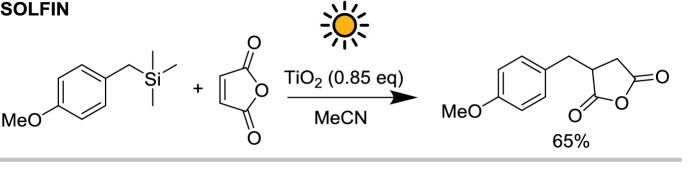
Solar light induced carbon–carbon bond formation via radical benzylation of electron-poor olefins

Similarly, in the SOLFIN, another light-limited photochemical reaction was performed by Albini and co-workers [22]. In this case, a titanium dioxide slurry was used as the photocatalyst to generate benzyl radicals from the corresponding 4-methoxybenzyl(trimethyl)silane (Scheme 5). To maintain a uniform suspension of the heterogeneous catalyst, a flux of nitrogen was mixed with the reaction mixture. Maleic anhydride and maleic acid were used as radical acceptor resulting in full conversion after 10 and 22 h of irradiation, respectively, on a 20 mmol scale. For the reaction with the anhydride, the product was obtained in 65% yield after recrystallization, resulting in a productivity of $6.5 \;{\text{mmol}}\,{\text{m}}^{{- 2}}\,{\text{h}}^{{- 1}}$. Apparent quantum yields of 1% (acid) and 3% (anhydride) were reported, thanks to the good transparency of the SOLFIN Pyrex tube to UV photons. Interesting, an apparent zero-order kinetic profile was observed, with the conversion correlating nicely with the integrated incident photon flux. Notably, given the recent advances in decarboxylative cross-coupling reactions, the succinic anhydride moieties resulting from the radical additions to maleic anhydride can be further functionalized, even in enantioselective ways [27].

### SOLARIS and PROPHIS

**Fig. 6:**
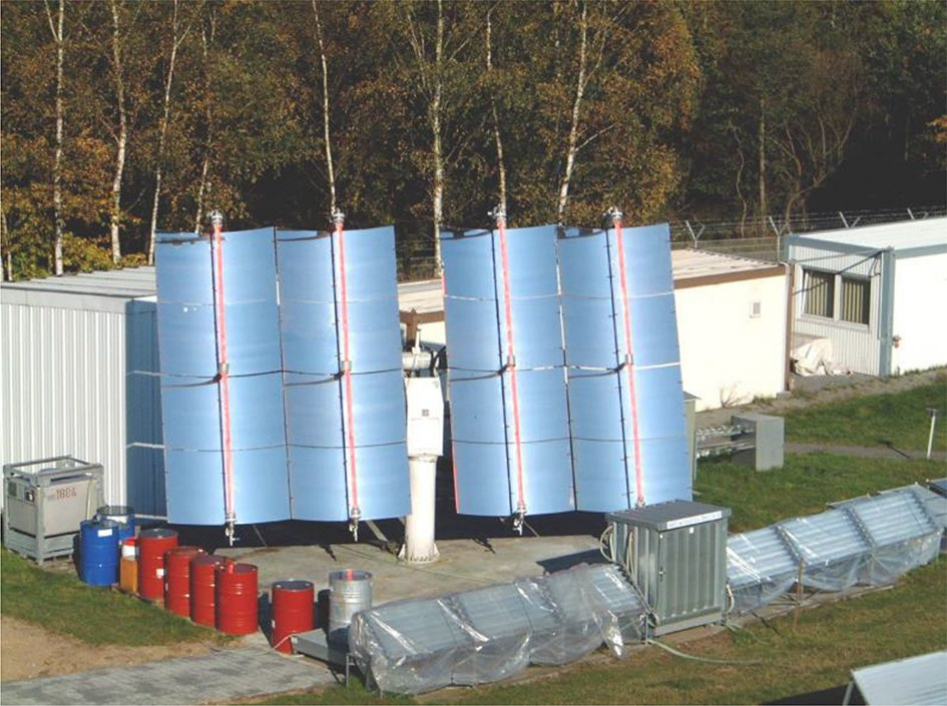
Photograph of the PROPHIS reactor at the DLR facility in Cologne. The four reaction tubes filled with a red reaction mixture and their parabolic collector are mounted on a Helioman solar tracking system

**Fig. 7:**
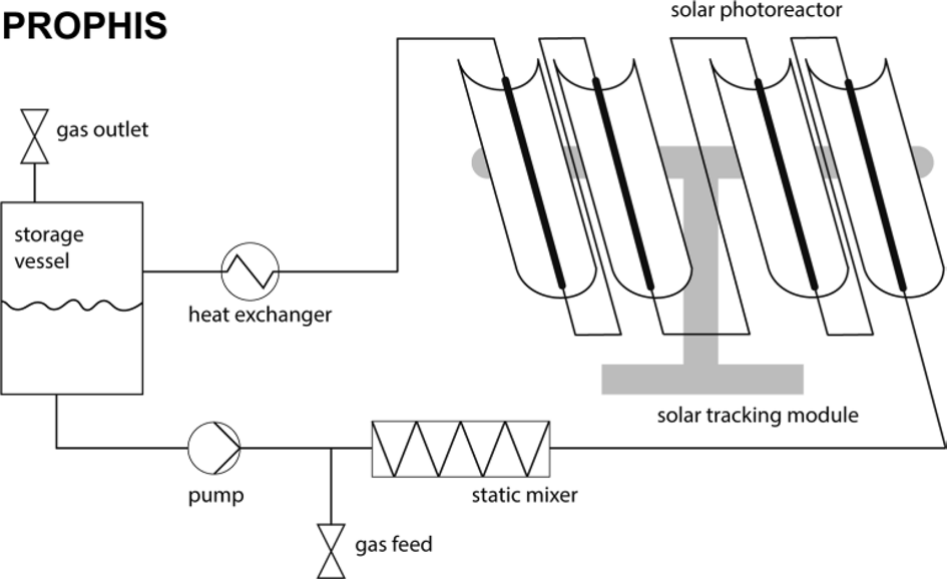
Schematic diagram of the PROPHIS reactor. The reaction is pumped from the storage vessel, optionally mixed with gas and continuously circulated in the photoreactor tubes. Notably, only a single gas–liquid mixer is present, explaining the lower efficiency of this design observed in photooxidations carried out with four apertures as opposed to a single one

A convincing example of how solar light can be exploited for the photochemical synthesis of fine chemicals on a large scale is constituted by the SOLARIS (solar photochemical synthesis of fine chemicals) reactor and its successor PROPHIS (parabolic trough-facility for organic photochemical syntheses), which are essentially the same device (see Fig. 6). The SOLARIS pilot experiment was jointly conducted at the Plataforma Solar de Almería (PSA) by the German Aerospace Center (DLR) and the Technical University of Aachen. In 1992, the reactor was dismantled and reassembled with some upgrades at the DLR research center of Köln-Porz (Germany). Compared to the SOLARIS, the PROPHIS was improved for what concerns the reflector material (Ag on glass vs. aluminum foil), the maximum reactor volume (from 70 to 120 l), while the total aperture (32 m^2^) and the geometric concentration ratio (32) both remain the same. The two reactors, therefore, share the same design. The reaction mixture is pumped through four Pyrex tubes placed in the focal point of four parabolic trough reflectors, mounted on a solar-tracking module (called Helioman). A gas-dosage inlet and a heat exchanger are also present in the recirculating loop to allow gas–liquid reaction and thermal control, respectively (see Fig. 7 for the complete flow scheme).

Because of its pilot experiment nature, the SOLARIS reactor was employed for several classical photochemical transformations, thus validating the potentiality of a solar-powered photoreactor. On the other hand, the PROPHIS has also been used for more innovative reactions.

**Scheme 6:**
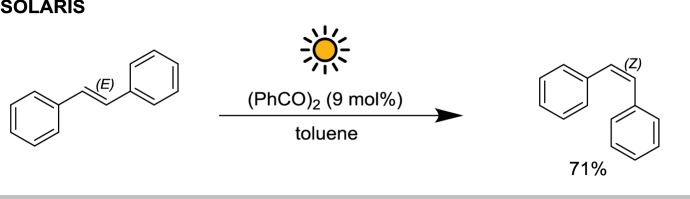
Benzil-catalyzed photoisomerization of trans-stilbene with solar light in the SOLARIS reactor

One of the classical photochemical reactions performed in the SOLARIS was the photoisomerization of *trans*-stilbene reported by Jung et al. [28]. Benzil was used as sensitizer and 3.4 kg of *cis*-isomer were obtained in 500 min starting from 4.8 kg of starting material in 85 L of toluene (71% isolated yield, 83% conversion), close to the photostationary equilibrium (Scheme 6). The productivity, in this case, was about $70 \;{\text{mmol}}\,{\text{m}}^{{- 2}}\,{\text{h}}^{{- 1}}$.

**Scheme 7:**
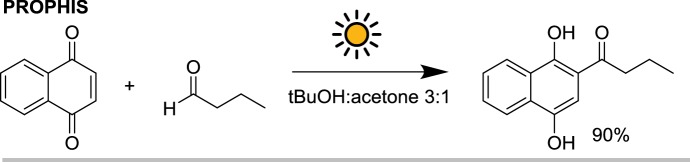
The photoacylation of naphthoquinone with butyraldehyde

Another large-scale experiment was performed at the PROPHIS by Mattay and co-workers [29]. In this case, three of the four troughs of the PROPHIS were used with 80 L of solvents for the photoacylation of 1,4-naphthoquinone with butyraldehyde on a 500 g (3.2 mol) scale (Scheme 7). The reaction took 24 h to reach full conversion (90% GC yield) over 3 days, only the first one of which in optimal weather conditions. Since for a solar reactor based on optical concentrators like the PROPHIS the direct irradiation constitutes the most important fraction of the global solar radiation, it was calculated that over 80% of the photons reaching the reaction in the 300–400 nm range over the 3 days were collected during the first day of irradiation.

**Fig. 8:**
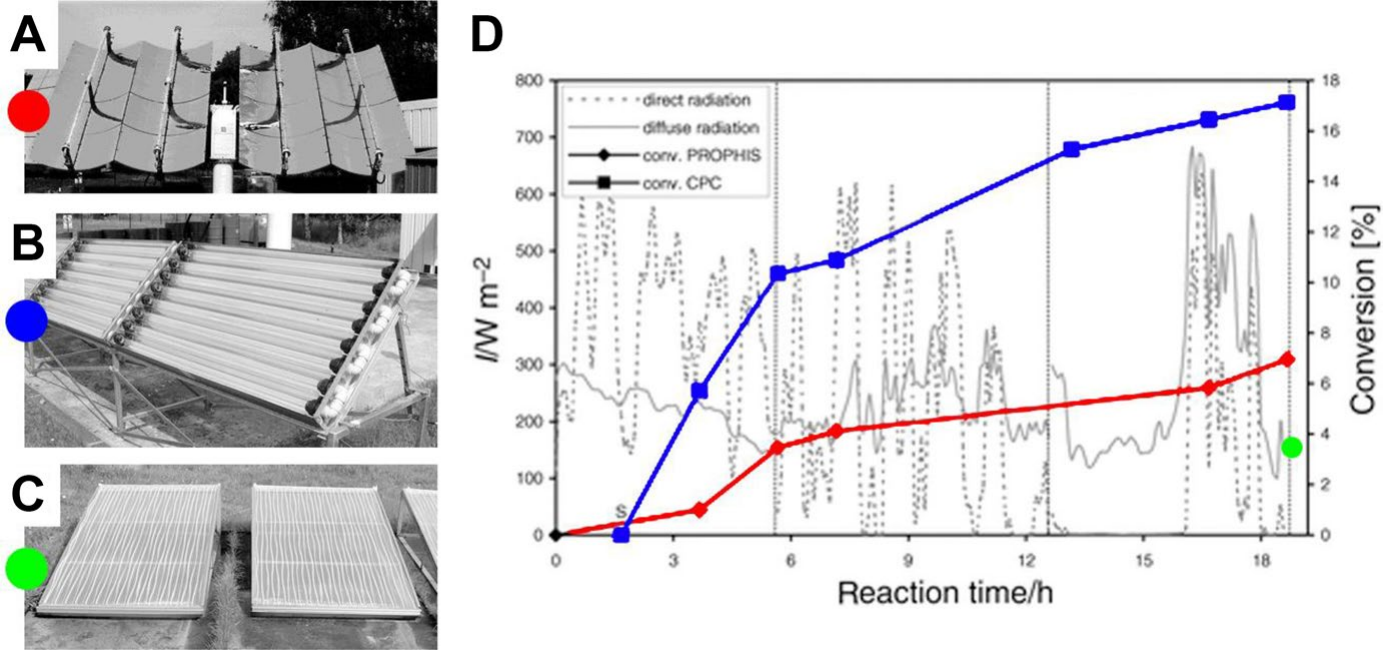
Different reactor designed compared in the photoacylation of 1,4-naphthoquinone with butyraldehyde. **a** PROPHIS (single module), **b** small CPC and **c** flat bed reactor, all having an irradiated area of 3 m^2^, **d** reaction conversion in the three reactors

Interestingly, in the same article, the PROPHIS reactor was compared with other two reactor designs with the same irradiated surface (3 m^2^): one based on smaller compound parabolic collectors, similar in design to the PROPHIS but with no solar tracking (concentrating factor ≈ 2–3) and a flat bed reactor (concentrating factor = 1, see Fig. 8 for a photograph of the three reactors). Among the three, the smaller CPC reactor exhibited the best performance thanks to its ability to concentrate both direct and a good portion of diffuse light. Notably, the ratio between the conversion in the CPC reactor and the PROPHIS (17% vs. 7%) matched the calculated ratio between the calculated amounts of photon collected by the two devices. Finally, the conversion in the flat bed was about half that in the PROPHIS (3.6% vs. 7%) partly due to the overnight solidification of the reaction mixture (containing *tert*-butanol), reducing the conversion in the following days.

**Scheme 8:**
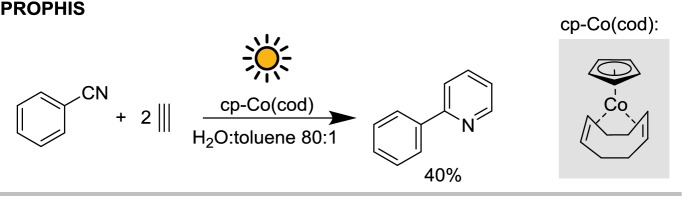
Solar cp-Co(cod) catalyzed [2 + 2 + 2] cycloaddition of acetylene to benzonitrile

Another reaction performed in the PROPHIS was the [2 + 2+2] cycloaddition of acetylene to benzonitrile, yielding the corresponding 2-phenylpyridine [30] (Scheme 8). Optimal results were obtained using a single trough module, presumably due to mass transfer limitation since the gas reactant is only added before the first module. Under optimized conditions, a conversion of 40.7% was achieved after 5.5 h on a 1.27 mol scale, with an isolated yield of 39.7% and the formation of just 1.3% of benzene byproduct. This corresponds to a productivity of $11.3 \;{\text{mmol}}\,{\text{m}}^{{- 2}}\,{\text{h}}^{{- 1}}$. Previously, the same group had already performed the same reaction with sunlight, on a smaller scale, with the SOLFIN reactor [31].

**Scheme 9:**
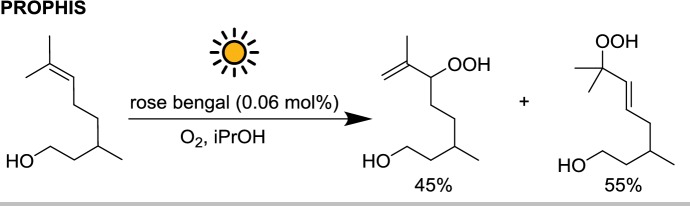
Single oxygen-mediated citronellol oxidation sensitized by rose bengal

The PROPHIS reactions previously described were mostly UV-driven. Since the highest intensity of solar radiation is observed in the visible range, it is expected that performing a photochemical reaction using visible light will result in faster reaction kinetics thanks to the higher photon flux. Indeed, when the PROPHIS was applied to the photooxygenation of citronellol sensitized by rose bengal, almost 2 L of starting material per hour could be converted to the corresponding hydroperoxide by using a single reactor channel (aperture 8 m^2^), with an remarkable productivity of ≈ $1.3 \;{\text{mol}}\,{\text{m}}^{{- 2}}\,{\text{h}}^{{- 1}}$ (Scheme 9) [32].

### MPI Line-focusing Reactor

**Scheme 10:**
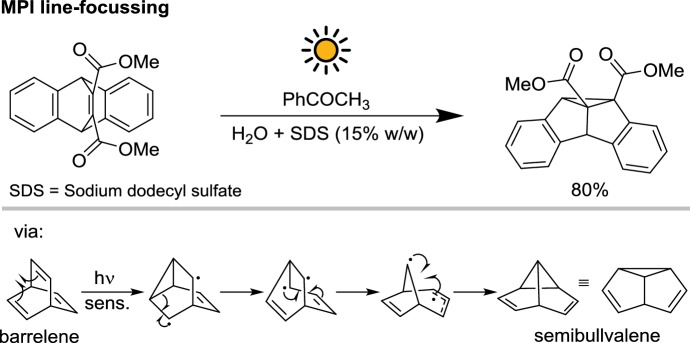
The di-π-methane rearrangements performed in the line focusing solar reactor at the MPI of Mülheim

**Fig. 9:**
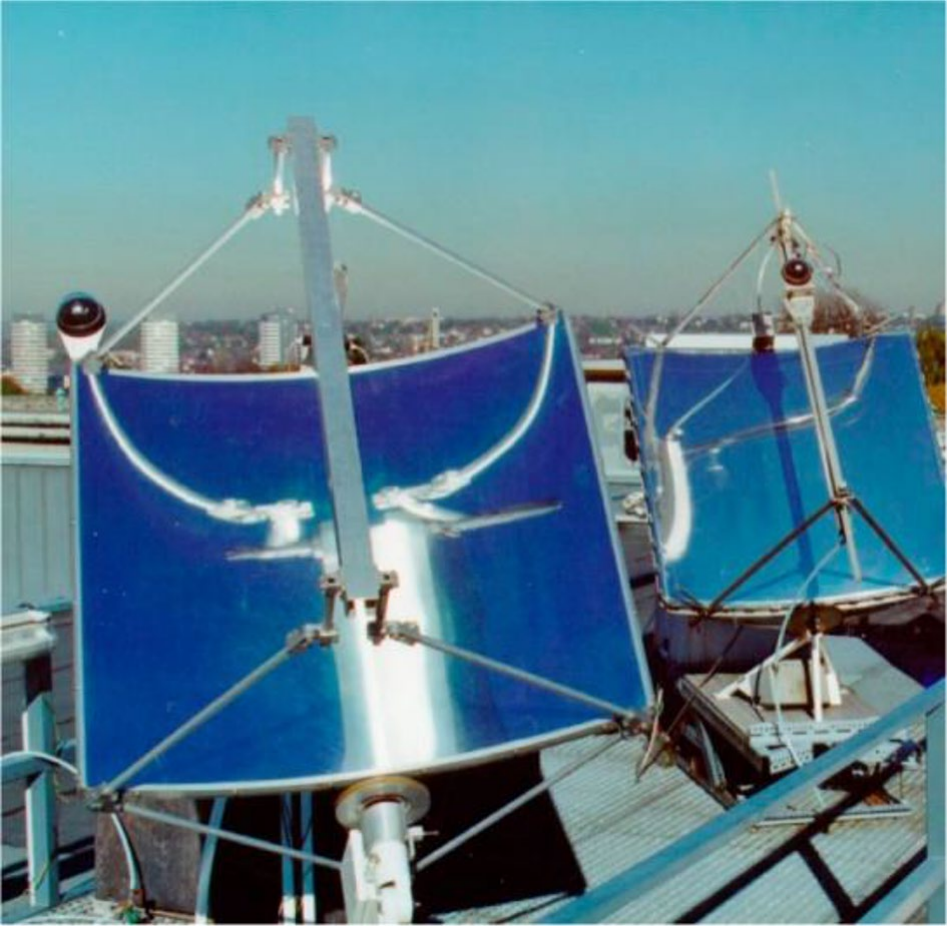
The line-focusing solar photoreactor at the Max Planck Institute of Mülheim. Reprinted with permission from [38]. Copyright 2016 American Chemical Society

A so-called “line focusing solar reactor” was employed between 1992 and 1997 at the Max Planck Institute (MPI) of Mülheim (Germany) for some di-π-methane rearrangements (Scheme 10) [33–35]. This solar reactor is an optically-concentrating design whose parabolic collector can be focused on the reaction vessel by applying vacuum to a chamber, resulting in the stretching of the aluminum film cover onto the parabolically-shaped plastic supporting frame (see Fig. 9). Due to the high concentration factor (up to 60 suns) a cooling tower was included in the design to cool the reaction.

Under optimized conditions, the barrelene derivative was exposed to solar light in a micellar solution of sodium dodecyl sulfate (SDS) in water in the presence of acetophenone as the photosensitizer. The semibullvalene product was formed in 80% isolated yield after 16 h of irradiation. Not surprisingly, the high optical concentration power of this reactor afforded a significant reduction of the reaction time, compared with the 104 h required for the direct excitation of the substrate. However, the performance of this reactor design is linked to the presence of clear sky: in partially sunny or a cloudy day, low-tech flat-bed collectors have shown to be superior thanks to their ability to efficiently collect the diffuse component of solar irradiance [36, 37].

### Sunflow and Similar Design

**Fig. 10:**
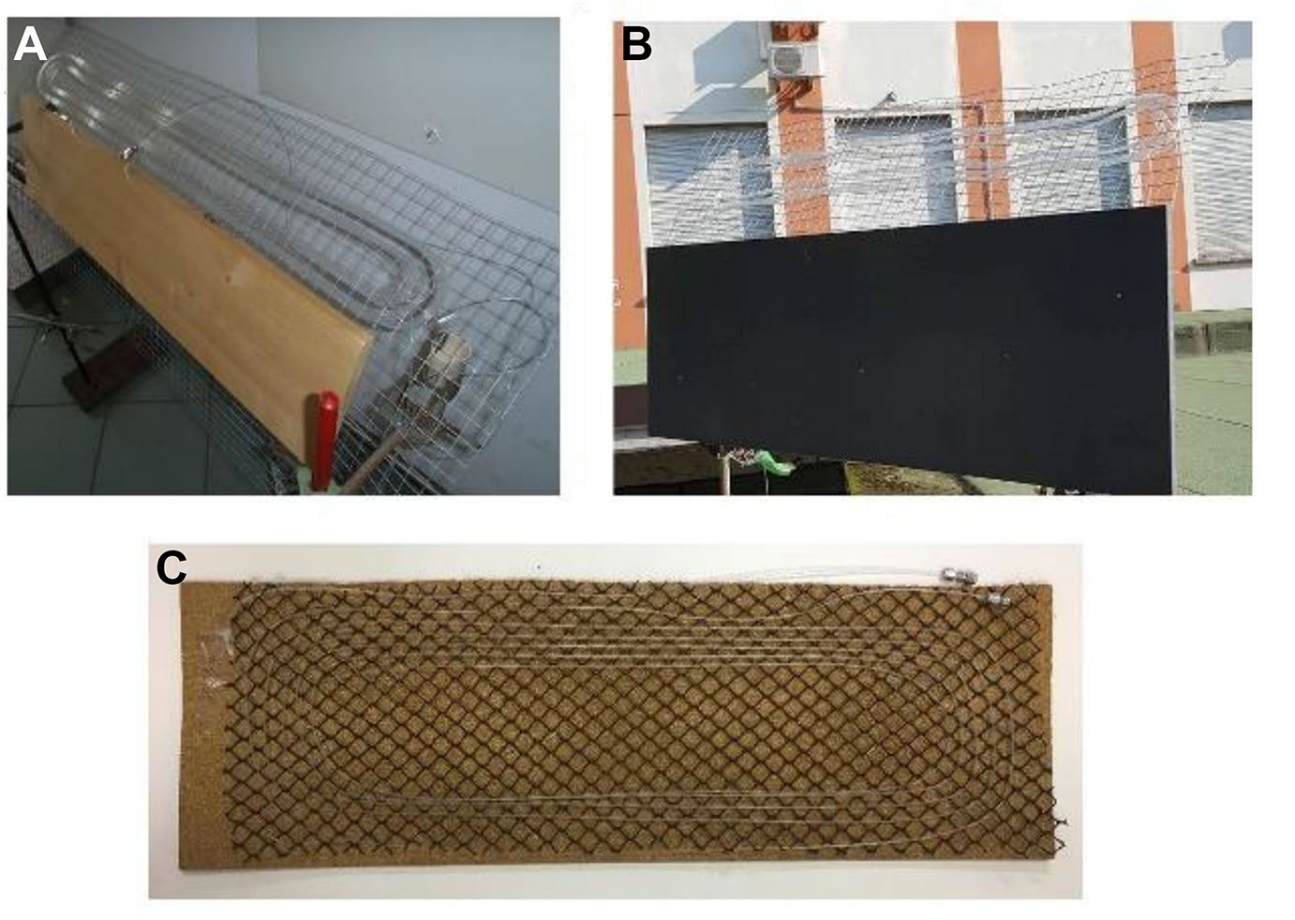
Photograph of the Sunflow reactors. **a** sunflow built in Mainz, Germany, **b** sunflow built in Ribeirão Preto, Brazil, **c** sunflow built in Pavia, Italy. Reprinted with permission from [39]. Copyright 2016 John Wiley and Sons

Recently, Opatz and co-workers reported a novel reactor for solar photoredox and H-atom transfer chemistry named “sunflow” [20]. Compared to the reactors previously described, the sunflow is simpler as it does not include any type of solar concentration. Furthermore, realizing the importance of a narrow residence time distribution to optimize the reaction time and prevent over-irradiation, the recirculating closed-loop design was replaced with a more efficient single-pass. The reactor is essentially constituted by a 25 m long FEP capillary (outer diameter 1.6 mm, inner diameter 1.0 mm) woven into an aviary fence (see Fig. 10). Thanks to the microflow size of the capillary used, a stable gas–liquid slug flow could be obtained, and the reactor was used in essentially the same configuration for both homogeneous and heterogeneous reactions, highlighting its versatility.

**Scheme 11:**
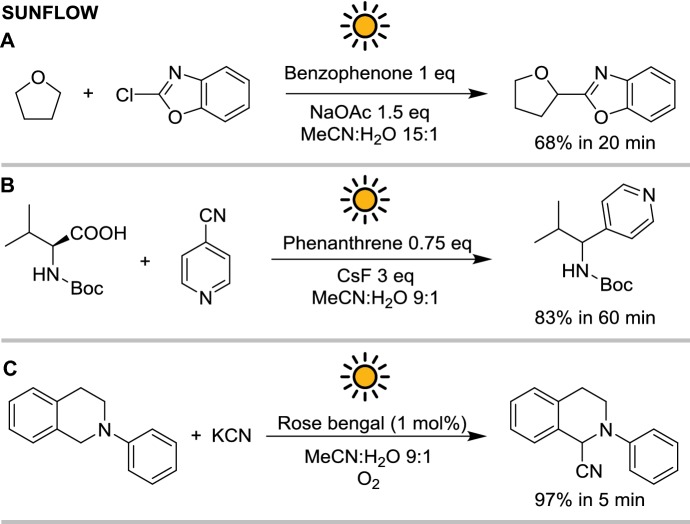
Reactions performed with sunflow: **a** 2-chlorobenzoxazole coupling, **b** Minisci-type coupling and **c** *N*-phenyl-tetrahydroisoquinoline cyanation

With this reactor, three different reactions were performed: (1) the benzophenone-mediated C–C coupling of 2-chlorobenzoxazoles with alcohols, ethers, and carbamates, (2) a phenanthrene catalyzed Minisci-type cross-coupling and (3) the oxidative α-cyanation of tertiary amines (see Scheme 11).

The sunflow is the first example of photoredox and H-atom transfer photoreaction performed in a microflow capillary powered by solar light. Initially, the reactor was used for the C–C coupling of 2-chloro benzazoles catalyzed by benzophenone, previously reported by the same group [40]. The reaction was significantly faster under solar irradiation than in the original batch protocol employing a 25 W UV-A lamp: full conversion was achieved in 20 min with solar irradiation versus 24 h with artificial lamps [40]. However, the little overlap between the solar spectrum and benzophenone absorption spectrum resulted in relatively slow reaction kinetics. Similarly, the UV-driven phenanthrene-catalyzed Minisci-type reaction of carboxylic acids with aromatic nitriles was performed with solar light resulting in an acceleration compared to the batch protocol employing artificial lamps but still requiring 60 min to reach full conversion [41]. Inversely, when a visible-light-absorbing photocatalyst was used in the α-cyanation of tertiary amines, full conversion was obtained between 5 and 10 min with just 1 mol % of catalyst loading. In particular, among the photocatalysts screened, the best results were obtained with rose bengal.

As observed for the PROPHIS, the absorption yield is often the main parameter dictating the reaction efficiency. While benzophenone and phenanthrene only marginally absorb in the UV-A, the strong absorption of rose bengal in the visible ($\lambda_{ \hbox{max} } = 558\; {\text{nm}}$) allows for faster reaction apparent kinetics with lower photocatalyst loading.

The sunflow reactor has also been applied by the same authors to the arylation of isonitriles and heteroaromatic substrates via photolysis of azosulfones [39].

**Scheme 12:**
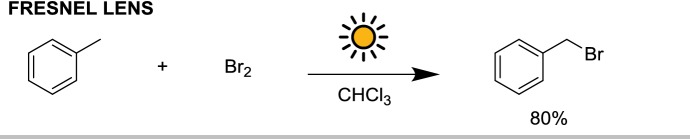
Benzylic mono-bromination under solar irradiation, concentrated via a Fresnel lens

A similar approach to flow solar photochemistry was reported by Kim et al. for the photo-induced benzylic bromination [42]. In this case, 5 m of FEP capillary were coiled and placed in a Dewar flask (diameter 10 cm) that serve as a reflector. On top of the Dewar, a 20 cm diameter Fresnel lens was used to concentrate solar light and direct it toward the capillary. Thanks to the good mixing behavior of the microreactor T mixer, good selectivity (up to 96% depending on the substrate) for the monobrominated species was possible. For the bromination of toluene, 90 s of sunlight irradiation were sufficient to afford an isolated yield of 80%, with a theoretical daily productivity of 44 grams (Scheme 12).

Finally, a non-concentrating setup essentially constituted by a quartz microreactor operated in recirculating fashion via a peristaltic pump was reported by Basheer and co-workers for the rose bengal mediated photooxygenation of furfural. In their simple reactor, the only stratagem specifically devised for solar irradiation was placing a mirror under the reactor to increase the reactor photon efficiency.

### Compound Parabolic Concentrator-based Reactors (CPC)

Compound parabolic concentrators (CPC) are essentially “round W” shaped reflectors generally used to focus solar light on a receiver tube. Since the diameter of the tube is often in the same order as magnitude of the reflector, small concentration factors are achieved by this design. Compared with the parabolic concentrators, no solar tracking is needed, making this solution simpler and more cost-efficient. However, CPC-based reactors are still characterized by some degree of optical concentration and a continuous-flow operation mode, making them superior to the flat bed design.

**Scheme 13:**
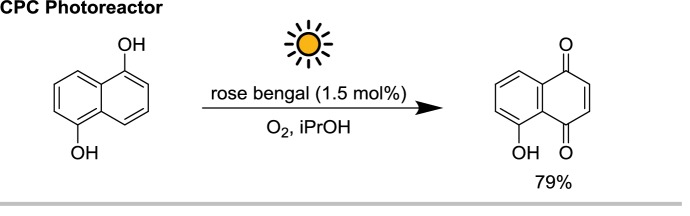
Solar singlet oxygen-mediated synthesis juglone from 5-hydroxy-1,4-naphthoquinone in a small-scale CPC reactor

Oelgemöller et al. used a small parabolic trough collector for the singlet oxygen-mediated synthesis of juglone from 5-hydroxy-1,4-naphthoquinone (Scheme 13) [32]. The parabolic collectors were covered with holographic mirrors whose reflectivity was centered on 550 nm ± 140 nm matching the absorption maximum of rose bengal, the photosensitizer used in the reaction. This approach circumvents the major limitation of optically concentrating photoreactors, which is the heating of the reaction mixture caused by the optical concentration of infrared photons.

**Fig. 11:**
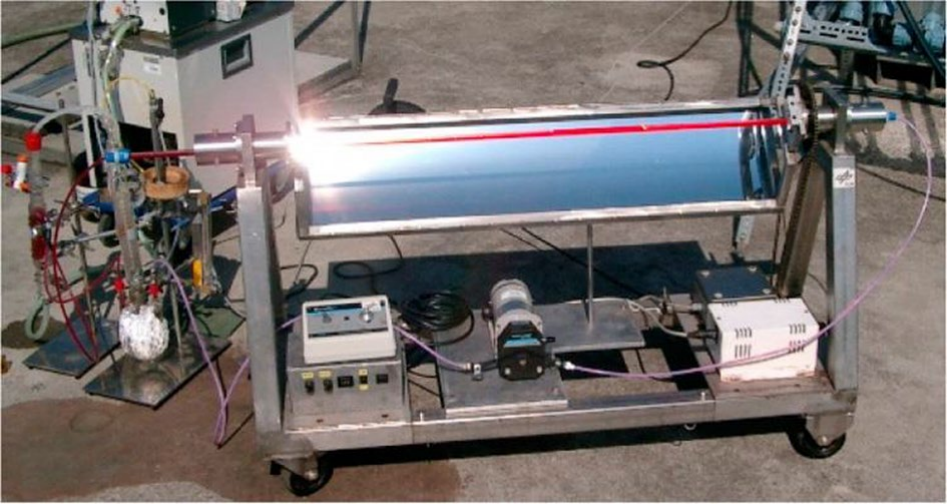
CPC reactor with holographic mirrors for the solar photooxygenation of 5-hydroxy-1,4-naphthoquinone, sensitized by rose bengal (as evident from the red color of the tube). Reprinted with permission from [38]. Copyright 2016 American Chemical Society

Despite the relatively small aperture of such a reactor (0.188 m^2^), 6.24 mmol of the substrate were converted in 9 h and a half of solar irradiation over 2 days, resulting in a productivity of about $3.5\; {\text{mmol}}\,{\text{m}}^{- 2}\,{\text{h}}^{ - 1}$. This value is significantly lower than the photooxygenation of citronellol performed in the PROPHIS reported in the same article, partly due to the lower reactivity of the substrate with singlet oxygen (see Fig. 11).

### Luminescent solar concentrator-photomicroreactor (LSC-PM)

**Fig. 12:**
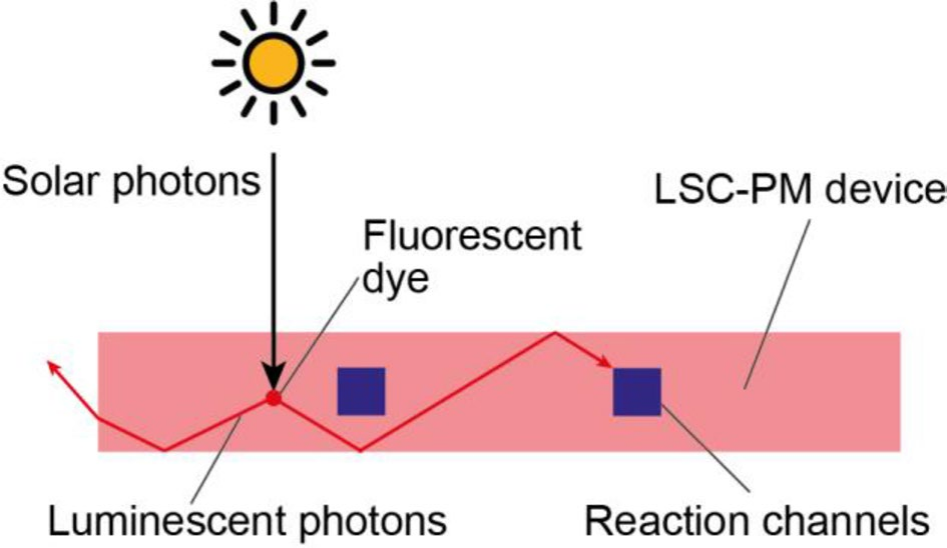
Working principle of the LSC-PM design. The solar photons reaching the device are absorbed by the fluorescent dye and re-emitted. The emitted photons are trapped in the polymeric material that acts as a waveguide and delivers the down-converted photons to the reaction channels

In 1994 Scharf and co-workers wrote that “Any industrial application must conform to the limitations imposed by the spectral distribution of the photons from the sun, the interruptions to the radiation due to the day/night rhythm, and the weather.” In this regard, the luminescent solar concentrator-photomicroreactor (LSC-PM) [18] provides an innovative solution to waive some of these requirements. While other solar photoreactors were designed to filter the solar spectrum and concentrate only the portion of radiation needed by the reaction (vide the holographic reflectors described before for some CPC reactors), the LSC-PM is the only reactor design that actively down-converts high-energy UV photons to longer wavelength, to match the reaction absorption requirements. This results in the deliberate modification of the solar spectrum to match the reaction absorption window, overcoming the limitation of the $\eta_{\text{Abs}}$ parameter in the expression of the total photon yield. The LSC-PM designed is based on an existing solar concentration technology, the luminescent solar concentrator (LSC) concept, embedded with a continuous-flow microreactor (see Fig. 12).

**Fig. 13:**
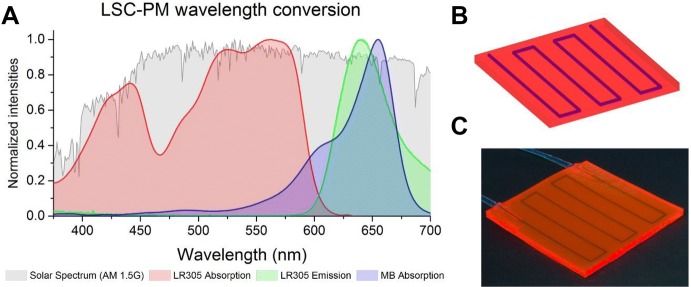
**a** The spectral conversion in the red LSC-PM, **b** a 3-D rendering of the device and **c** a photograph of the reactor

Luminescent solar concentrators are glass or polymeric slabs doped with a luminophore, generally a fluorescent dye. The photons absorbed by the dye are reemitted via fluorescence and have a high probability of being trapped in the slab due to total internal reflection. The whole slab acts therefore as a light guide. When a flow reactor is integrated with this design, the photons generated in the LSC can be used to power a photochemical reaction (see Fig. 13).

**Scheme 14:**
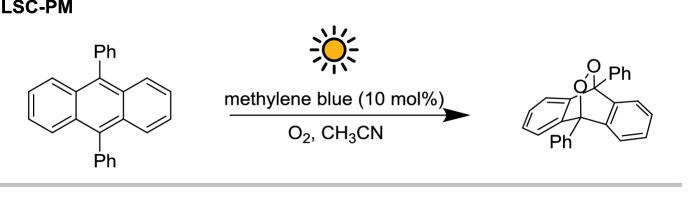
The photooxygenation of 9,10-diphenylanthracene, a reaction whose apparent kinetics is light-limited, has been used as a probe to characterize the LSC-PM light-harvesting efficiency

The LSC-PM design, introduced by Nöel, Debije, and co-workers [18], can be manufactured with different dyes, as far as the absorption of the fluorophore matches with the spectral demands of the photochemical reaction being performed. In its first version, the LSC-PM was a 150 uL flow reactor embedded in a 5 × 5 × 0.3 cm^3^ polydimethylsiloxane (PDMS) slab doped with a red fluorescent dye. The wavelength of the luminescent photons generated matched the absorption of the photosensitizer used for the model reaction (methylene blue). In particular, the singlet oxygen-mediated photooxygenation of 9,10-diphenylanthracene (DPA) was chosen since its kinetic profile is light-limited (Scheme 14). Therefore, the increased photon flux received by the reaction mixture translated in a four-fold acceleration to the reaction rate. It was shown that such acceleration is due to both the wavelength down-conversion and the concentrating characteristic of the LSC device.

**Fig. 14:**
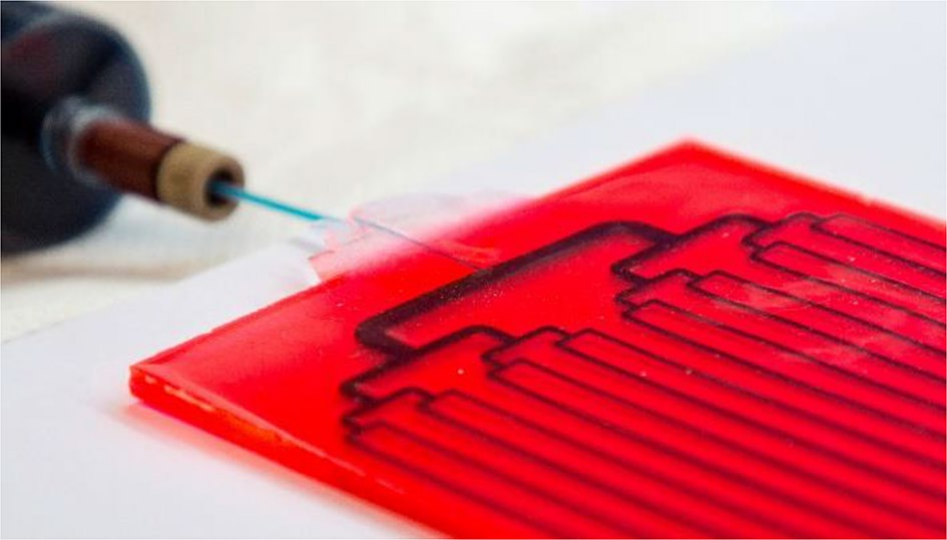
The scaled-up version of the LSC-PM reactor with 16 channels and a bifurcation design distributor

Once the LSC-PM design was validated with outdoor experiments, the same group developed a scaled-up version of the reactor to increase the productivity [43]. Generally, one of the advantages of flow chemistry is the straightforward scaling up by numbering up. With this regard, the most efficient approach is undoubtedly an internal numbering-up strategy, where a single pump is connected to multiple reaction channels via a distributor [44]. When this approach was adopted for the LSC-PM reactor, however, the inter-channel spacing became a crucial aspect since the lightguide has the function of harvesting the photons for the neighboring channels (see Fig. 14). After a screening of different reactor designs, an optimal spacing of 2.5 cm was chosen and reactors were manufactured and tested, resulting in a performance similar to the original design but with an improved productivity [43].

**Fig. 15:**
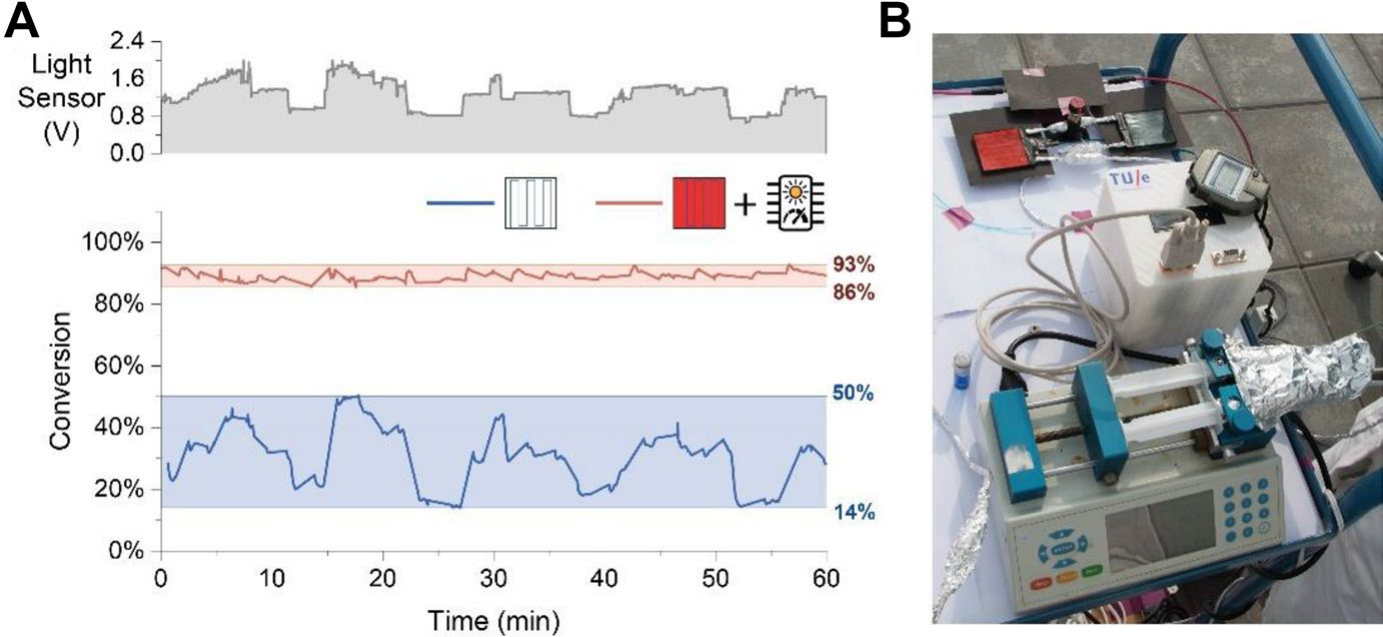
**a** Reaction conversion comparison between an LSC-PM reactor connected to the reaction control system and a non-LSC reactor. While the conversion in the traditional reaction (blue line) follows the variation in solar irradiance (graph in grey), the LSC-PM reactor (red line) connected to the microcontroller exhibits stable reaction performance. **b** The setup used for the sunlight experiments

To improve the understanding on the reactor photophysics, and aid in the development of other reactors based on the LSC-PM concept, a detailed description of the photon path within the device was later reported by the same group via Monte Carlo ray-tracing simulations [45]. In this analysis, it was observed that the photon flux emitted at the device edges is proportional to that witnessed by the reaction mixture flowing in the reactor channel (see Fig. 12). Based on this key observation, Noël and co-workers rationalized that it would have been possible to acquire accurate information on the instant photon flux reaching the reaction channel by just monitoring the variation in edge emitted photons. Once the relationship between the kinetic profile and the light intensity is known, this information can then be used to compensate the variations in solar irradiance by varying the residence time in the reactor, affording a constant reaction conversion [46]. A simple reaction control system was therefore designed by the same group that updated in real-time the residence time in the reactor by modifying the pump flow rate based on the light intensity measured via a phototransistor placed at the device edge. After a calibration of the reaction system, steady conversions were obtained even in fluctuating solar irradiance conditions (see Fig. 15). This proof of concept is extremely significant as it allows addressing a long-standing issue in solar photochemistry, namely, the possibility of having a continuous process powered by a fluctuating energy input.

### Outlook

From the different articles reported earlier in the chapter, it is clear how solar photochemistry is progressively adopting simpler yet more advanced and photon-efficient reactor designs (e.g., replacing active solar-tracking with holographic reflectors or LSC-based concentrators). While low density and intermittent availability are well-known limitations of solar radiation [47], technological solutions are now existing to mitigate or overcome those well-known limitations, like the reaction control module described above. These recent developments have reduced the distance for the adoption of solar photochemistry in the production of chemicals. Another potential application of solar photochemistry could be the photocatalytic lignin-depolymerization, creating a renewable approach to bio-based chemicals [48].

In the absence of subsidies directly promoting solar photochemistry, it is likely that the first industrial applications will be specialty chemicals having niche markets and high profit margins. With further development in the reactor efficiencies, it is expected that in the future the solar manufacturing of fine chemicals could be economically competitive for several fine and specialty chemicals. Nowadays, specialty chemicals are usually produced batchwise as opposed to the continuous process that characterizes most bulk chemicals. Since efficient solar photochemistry is inextricably linked with a flow operation mode, the barrier for adoption is currently significant as both a batch-to-flow and a lamp-to-solar conversion are needed. The reluctance to change of the chemical industry is well exemplified by economic evaluation of the industrial synthesis of ε-caprolactam via solar photooximation of cyclohexane, which already in 1999 had shown that the return of investment for the solar photochemical process is superior to the existing lamp-driven approach [49, 50]. Nevertheless, no solar-powered power plants that we are aware of have been commissioned or even planned so far. We hope that future research in simpler, more efficient and versatile reactor design, coupled with the growing interest toward visible-light photochemistry, can change this situation in the future and unleash the sustainable potential of solar energy for the production of chemicals.
